# Inefficient recruitment of kinesin-1 to melanosomes precludes it from facilitating their transport

**DOI:** 10.1242/jcs.186064

**Published:** 2017-06-15

**Authors:** Christopher L. Robinson, Richard D. Evans, Deborah A. Briggs, Jose S. Ramalho, Alistair N. Hume

**Affiliations:** 1 School of Life Sciences, University of Nottingham, Nottingham, NG7 2UH, UK; 2 CEDOC Faculdade de Ciencias Medicas, Universidade Nova de Lisboa, 1169-056 Lisbon, Portugal

**Keywords:** Organelle transport, Kinesin-1, Myosin-Va, Actin, Microtubules, Melanocyte

## Abstract

Microtubules and F-actin, and their associated motor proteins, are considered to play complementary roles in long- and short-range organelle transport. However, there is growing appreciation that myosin/F-actin networks can drive long-range transport. In melanocytes, myosin-Va and kinesin-1 have both been proposed as long-range centrifugal transporters moving melanosomes into the peripheral dendrites. Here, we investigated the role of kinesin-1 heavy chain (Kif5b) and its suggested targeting factor Rab1a in transport. We performed confocal microscopy and subcellular fractionation, but did not detect Kif5b or Rab1a on melanosomes. Meanwhile functional studies, using siRNA knockdown and dominant negative mutants, did not support a role for Kif5b or Rab1a in melanosome transport. To probe the potential of Kif5b to function in transport, we generated fusion proteins that target active Kif5b to melanosomes and tested their ability to rescue perinuclear clustering in myosin-Va-deficient cells. Expression of these chimeras, but not full-length Kif5b, dispersed melanosomes with similar efficiency to myosin-Va. Our data indicate that kinesin and microtubules can compensate for defects in myosin-Va and actin-based transport in mammals, but that endogenous Kif5b does not have an important role in transport of melanocytes due to its inefficient recruitment to melanosomes.

## Abstract (precis)

**Highlighted Article:** We show that Kif5b can compensate for defects in myosin-Va-based transport in mammals, but that endogenous Kif5b plays a minimal role in transport in melanocytes due to inefficient recruitment to melanosomes.

## INTRODUCTION

Actin and microtubule (MT) cytoskeleton tracks and their associated motor proteins regulate intracellular organelle transport in eukaryotes (Alberts et al., 2008; Barlan et al., 2013; Ross et al., 2008). MTs emanate from the centrosome, or MT-organising centre, with their fast-growing ‘plus’ ends oriented towards the plasma membrane and their slow-growing ‘minus’ ends focused at the centrosome. Thus minus-end-directed dynein and plus-end-directed kinesin motors can transport cargo over long distances (>1 µm) along these tracks (Hirokawa et al., 2009; Kardon and Vale, 2009). In contrast, the actin cytoskeleton in animal cells typically appears to consist of a complex network of short (<1 µm) randomly oriented filaments, suggesting that myosin motors tether or move cargo only short distances (Woolner and Bement, 2009; Wu et al., 1998). These observations are the cornerstone of the ‘highways and local roads’ model for transport along MTs and actin tracks (Woolner and Bement, 2009). Previously, this model has been supported by studies of the transport of various intracellular cargoes, including melanosomes in mouse melanocytes (Ross et al., 2008; Goode et al., 2000; Hume et al., 2011; Fukuda, 2013; Hammer and Sellers, 2012). Elegant studies that used the FKBP–rapamycin–FRB interaction system (where FKBP is FK506-binding protein and FRB is FKBP/rapamycin-binding protein) to specifically recruit myosin, kinesin and dynein motors to peroxisomes in COS-7 cells came to similar conclusions, i.e. MT motors drive long-range organelle transport from the cell centre to the periphery, while myosin motors move cargo locally at the cell periphery (Barlan et al., 2013; Goode et al., 2000; Kapitein et al., 2013; Ross et al., 2008). Nevertheless, there is also evidence from several systems that actin and myosin networks can drive organelle transport and positioning over long distances in the absence of MTs. For instance, in yeast, Myo2 transports cargo, including vacuoles and secretory vesicles, along actin bundles into the bud (Pruyne et al., 2004), while in murine oocytes, endosomes organise a network of actin filaments that allow myosin-Vb-dependent transport to the plasma membrane (Schuh, 2011; Holubcova et al., 2013).

Skin melanocytes reside in the hair follicles and the basal layer of the epidermis. There they synthesise pigment in lysosome-related organelles, termed melanosomes, which they then distribute to neighbouring keratinocytes via a network of dendrites (Fukuda, 2013; Hume et al., 2011; Raposo and Marks, 2007). Early studies revealed that the dendrites of melanocytes derived from the myosin-Va-null (dilute) mouse were devoid of pigment, suggesting that myosin-Va has a role in transporting melanosomes into dendrites (centrifugal transport) or tethering them there (Provance et al., 1996; Wei et al., 1997). Later studies revealed that a subpopulation of melanosomes in dilute melanocytes undertake bi-directional transport along MTs that extend along the length of the dendrites. However, this transport was insufficient to allow peripheral accumulation of melanosomes, suggesting that myosin-Va tethers (or ‘captures’) melanosomes at the dendrite tip by attaching them to randomly oriented actin filaments and prevents them from returning to the cell body (Wu et al., 1998). This ‘co-operative capture’ model for melanosome transport proposed that melanosomes accumulate in dendrites by sequential long-range transport along MTs, and myosin-Va- and actin-dependent tethering at the periphery. Follow-up studies revealed that the small GTPase Rab27a and its effector melanophilin recruit and activate myosin-Va at the melanosomes (Wu et al., 2002; Hume et al., 2001; Fukuda et al., 2002). In line with the co-operative capture model, other studies carried out at that time also suggested that kinesin-1 and cytoplasmic dynein associate with melanosomes and drive their MT-dependent movement and transfer to keratinocytes (Vancoillie et al., 2000a,b; Byers et al., 2000; Hara et al., 2000). More recent studies have revisited this topic and have proposed that melanosomal recruitment of dynein and dynactin is regulated by Rab36, melanoregulin and the Rab7 effector protein Rab-interacting lysosomal protein (RILP) (Matsui et al., 2012; Ohbayashi et al., 2012).

We previously tested the co-operative capture model by directly examining the role of MTs, F-actin and myosin-Va in melanosome transport (Evans et al., 2014). We found that, although video-microscopy reveals that ∼10% of melanosomes in wild-type melanocytes move bi-directionally on MTs at steady-state, MT integrity was only essential for centripetal (towards the cell body), and not centrifugal, transport (Evans et al., 2014; Hume et al., 2011). Instead, we found that centrifugal transport was driven by myosin-Va working in concert with a pool of dynamic F-actin, and that this process was accelerated in cells depleted of MTs, suggesting that MT and F-actin transport mechanisms oppose one another. Moreover isoform-specific adaptations, e.g. lever arm length and dynamic interaction with F-actin, which allow myosin-Va to move processively towards the plus-ends (barbed ends) of F-actin *in vitro*, were essential for its function in melanocytes. These observations indicate that myosin-Va is a transporter that works with a dynamic F-actin network, polarised towards the cell membrane, to move melanosomes to the cell periphery, and not a tether as previously suggested (Wu et al., 1998). As MTs are tracks for kinesin and dynein motors, our data also support an essential role for cytoplasmic dynein in centripetal transport but not kinesin-1, or other plus-end-directed motors, in centrifugal transport.

In contrast, two recent reports proposed that the small GTPase Rab1a [previously shown to have a highly conserved function in endoplasmic reticulum (ER)-to-Golgi transport; Tisdale et al., 1992; Segev et al., 1988] can recruit the kinesin-1 heavy chain Kif5b to melanosomes, via its effector SKIP (SifA- and kinesin-interacting protein; also known as PLEKHM2, pleckstrin homology domain-containing family M2) and kinesin light chain 2 (KLC2), and thereby regulate MT-dependent centrifugal melanosome transport (Ishida et al., 2012, 2015). Consistent with this hypothesis these studies showed that siRNA knockdown of these proteins individually resulted in perinuclear melanosome clustering, although in only 20–40% of transfected cells. Meanwhile, co-expression of the constitutively active Rab1a mutant (Rab1a^Q70L^) and SKIP resulted in peripheral accumulation of melanosomes in 25% of cells. Overall, these observations provide some support to the idea that Kif5b might be a driver of centrifugal melanosome transport.

To try to resolve this controversy, here, we tested head-on the role of Kif5b and Rab1a in melanosome transport by investigating their localisation and function in melanocytes. Our results indicate that neither protein is enriched at the melanosome membrane and that neither plays a detectable role in melanosome transport. By contrast, we found that fusion proteins that forcibly direct active Kif5b to melanosomes can efficiently disperse clustered melanosomes along MTs in myosin-Va-deficient melanocytes. We suggest that myosin-Va, and not Kif5b, is the dominant centrifugal transport protein in melanocytes, and that the limited capacity of melanosomes to recruit Kif5b restricts its function in this process.

## RESULTS

### The distribution of Kif5b and Rab1a within melanocytes is inconsistent with their proposed role in melanosome transport

**Fig. 1.**
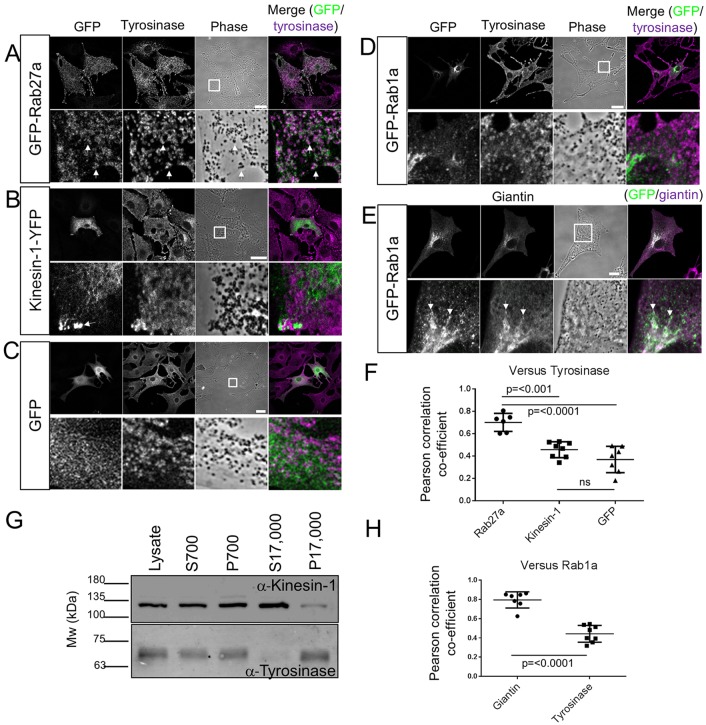
**The intracellular distribution of Kif5b and Rab1a in mouse melanocytes.** melan-a cells were transiently transfected with plasmid vectors allowing the expression of the indicated GFP or YFP fusion proteins. Cells were fixed after 48 h, stained with the indicated organelle marker-specific antibodies, and the intracellular distribution of GFP or YFP and organelle markers was observed using a confocal microscope (as described in the Materials and Methods). (A–E) Single confocal *z*-sections showing the distribution of GFP–Rab27a (A), Kif5b–YFP (B), GFP (C) and GFP–Rab1a (D,E) relative to organelle markers. Tyrosinase marks melanosomes (A-D) and giantin marks the Golgi apparatus (E), respectively. Phase-contrast and transmitted-light images show the distribution of pigmented melanosomes. In merge images, GFP is coloured green and organelle markers are coloured magenta; thus, white pixels indicate colocalisation. The white squares in the phase images indicate the part of the cell that is presented in the high-magnification panels below. Arrows in A and E highlight areas of colocalisation, while the arrows in B indicate the accumulation of Kif5b–YFP at the peripheral plus-tips of MTs. Scale bars: 20 µm. (F,H) Scatter plots showing the extent of colocalisation (measured using the PCC) between tyrosinase and Rab27a (*n*=6), Kif5b (*n*=8) and GFP (*n*=7) (F), and between GFP-Rab1a and tyrosinase (*n*=8) or giantin (*n*=7) in melan-a cells (H). Horizontal bars show the median and 25th and 75th percentile of each population. *P*-values were determined by one-way ANOVA for differences in PCC values (ns, not significant). (G) Western blot showing the expression of Kif5b and tyrosinase in subcellular fractions of melan-a cells (as described in Materials and Methods).

As a first step to investigate the possible role of Kif5b and Rab1a in centrifugal melanosome transport, we investigated their intracellular localisation in melanocytes. To do this, we transiently expressed GFP–Rab1a and Kif5b–YFP fusion proteins in immortal wild-type mouse melanocytes (melan-a) and then used confocal immunofluorescence microscopy (CIFM) to examine their localisation relative to melanosomes. Previous studies found that GFP–Rab1a could rescue Golgi fragmentation in HeLa cells depleted of endogenous Rab1a (Aizawa and Fukuda, 2015). Meanwhile, Kif5b–YFP had the same localisation as MT1-MMP (also known as MMP14), whose surface expression is regulated by Kif5b, in MDA-MB-231 cells (Marchesin et al., 2015). These observations indicate that these fusion proteins retain function. In contrast to GFP–Rab27a, neither Kif5b–YFP nor GFP–Rab1a was enriched in areas containing melanosomes, as detected using phase-contrast microscopy and anti-tyrosinase staining (Fig. 1A,B,D). Instead, much of the Kif5b–YFP, like GFP alone, appeared to be distributed uniformly throughout the cytoplasm with a subset accumulated in peripheral spots that are likely to correspond to the fast-growing plus-tips of MTs, which are located in dendrite tips (Fig. 1B,C) (Hume et al., 2007; Wu et al., 2005). This is consistent with the ability of Kif5b motor protein to move processively towards the plus-ends of MTs (Hirokawa et al., 2009). In line with these observations, colocalisation analysis revealed no significant difference in the overlap between tyrosinase and Kif5b–YFP or GFP alone [mean±s.d. Pearson's correlation coefficient (PCC)=0.457±0.07125 for Kif5b–YFP versus 0.3691±0.1183 for GFP]. In contrast, and consistent with its role in melanosome transport, Rab27a exhibited a significantly higher level of colocalisation with tyrosinase (mean±s.d. PCC=0.701±0.084; Fig. 1F).

In parallel, we used immunoblotting to test the relative abundance of endogenous Kif5b in a melanosome-enriched (P17,000) fraction of melan-a cells, using Kif5b-specific antibodies. Blotting using anti-tyrosinase antibodies, and comparison of the intensity of bands in the P17,000 and S17,000 fractions confirmed melanosome enrichment in the P17,000 fraction relative to the S17,000 fraction. Meanwhile comparison of the intensities of Kif5b-specific bands in the P17,000 and S17,000 fractions revealed the opposite, i.e. that Kif5b was enriched in a fraction containing low-density organelles and cytosol (S17,000) and not in the tyrosinase- and melanosome-enriched P17,000 fraction. The reciprocal intensities of the bands for Kif5b and tyrosinase in these fractions indicates that, at most, only a small fraction of Kif5b associates with melanosomes at steady state (Fig. 1G).

CIFM revealed that GFP–Rab1a was mostly distributed in the perinuclear cytoplasm, consistent with its highly conserved role in regulating ER-to-Golgi transport (Fig. 1D,E) (Tisdale et al., 1992; Nuoffer et al., 1994). In support of this, we found that the intracellular distribution of GFP–Rab1a correlated significantly more closely with that of endogenous giantin, a Golgi matrix protein, than tyrosinase (mean±s.d. PCC=0.795±0.084 for giantin, versus 0.443±0.087 for tyrosinase; Fig. 1E,H) (Linstedt and Hauri, 1993).

Thus, our data indicate that Rab1a and Kif5b are unlikely to strongly associate with melanosomes in melanocytes. Given that proteins, e.g. Rab27a and myosin-Va, which have well-established roles in regulating melanosome transport, colocalise with these organelles (Fig. 1F) (Hammer and Sellers, 2012; Hume and Seabra, 2011), the lack of apparent association between melanosomes, Rab1a and Kif5b argues against a direct role for these proteins in melanosome transport. In line with this, we found that a motor-less version of Kif5b (GFP–Kif5bΔ350) distributed in a filament-like pattern throughout the cytoplasm (that may correlate with MTs), while Myc-tagged SKIP accumulated in the peripheral cytoplasm, consistent with its previously reported Kif5b-dependent dispersion (Rosa-Ferreira and Munro, 2011; Navone et al., 1992). Thus, neither displayed obvious colocalisation with melanosomes (Fig. S1A).

### Functional disruption of Kif5b affects centrifugal transport of mitochondria, but not melanosomes, in melanocytes

**Fig. 2.**
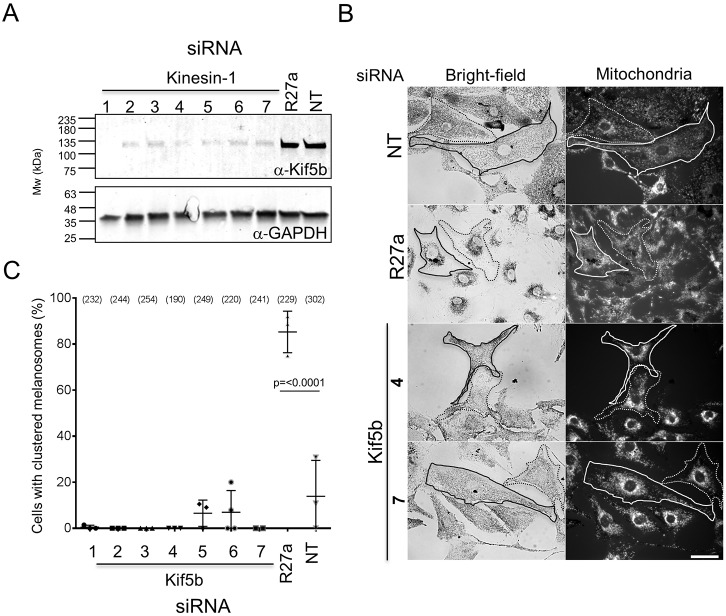
**Knockdown of Kif5b in melanocytes affects the distribution of mitochondria but not melanosomes.** melan-a cells were transfected with the indicated siRNA, and protein levels and melanosome clustering were measured (as described in Materials and Methods). (A) Western blot showing the level of Kif5b and GAPDH (loading control) proteins in lysates of siRNA-transfected cells. R27a, Rab27a. The blot displayed is representative of three independent blots from three independent transfections. (B) Representative bright-field and fluorescence images (left and right panels) showing the subcellular distribution of melanosomes and mitochondria, respectively, in fields of siRNA-transfected melanocytes. Traces indicate cell borders to highlight clustering of mitochondria in Kif5b versus Rab27a and control siRNA-transfected cells. Scale bar: 20 µm. (C) A scatter plot showing the percentage of melanocytes manifesting perinuclear clustered melanosomes for each siRNA. Data are from the independent transfections each performed in triplicate on different pools of cells. Plotted points represent the mean percentage of cells with perinuclear clustered melanosomes from each experiment (as described in the Materials and Methods). Bracketed numbers indicate the total number of cells analysed. Bars show the median and 25th and 75th percentile of each population.

To test the function of Kif5b in transport, we used siRNA to deplete Kif5b in melanocytes and then examined the effects of this on two read-outs of centrifugal transport: (1) maintenance of dispersed melanosome distribution in melan-a cells, and (2) myosin-Va-dependent dispersion of perinuclear melanosome clustering in myosin-Va-null (melan-d1) cells. Western blotting confirmed that several pairs of siRNA oligonucleotides were effective in depleting Kif5b protein (Fig. 2A). [These siRNA included sequences used previously to deplete these targets (Ishida et al., 2012, 2015; Gupta et al., 2008; see Materials and Methods for details)].

In the first centrifugal transport assay (maintenance of dispersed melanosome distribution in melan-a cells), control siRNA depletion of Rab27a resulted in perinuclear melanosome clustering in a significant majority of melan-a cells [Fig. 2B,C; cells with clustered melanosomes=85.26±9.038% for Rab27a, versus 13.88±15.60% for non-targeted (NT) control siRNA transfected cells; mean±s.d.], while Kif5b-specific siRNA did not. This indicates that, unlike Rab27a and its effectors (melanophilin and myosin-Va), Kif5b does not play a significant role in centrifugal transport.

**Fig. 3.**
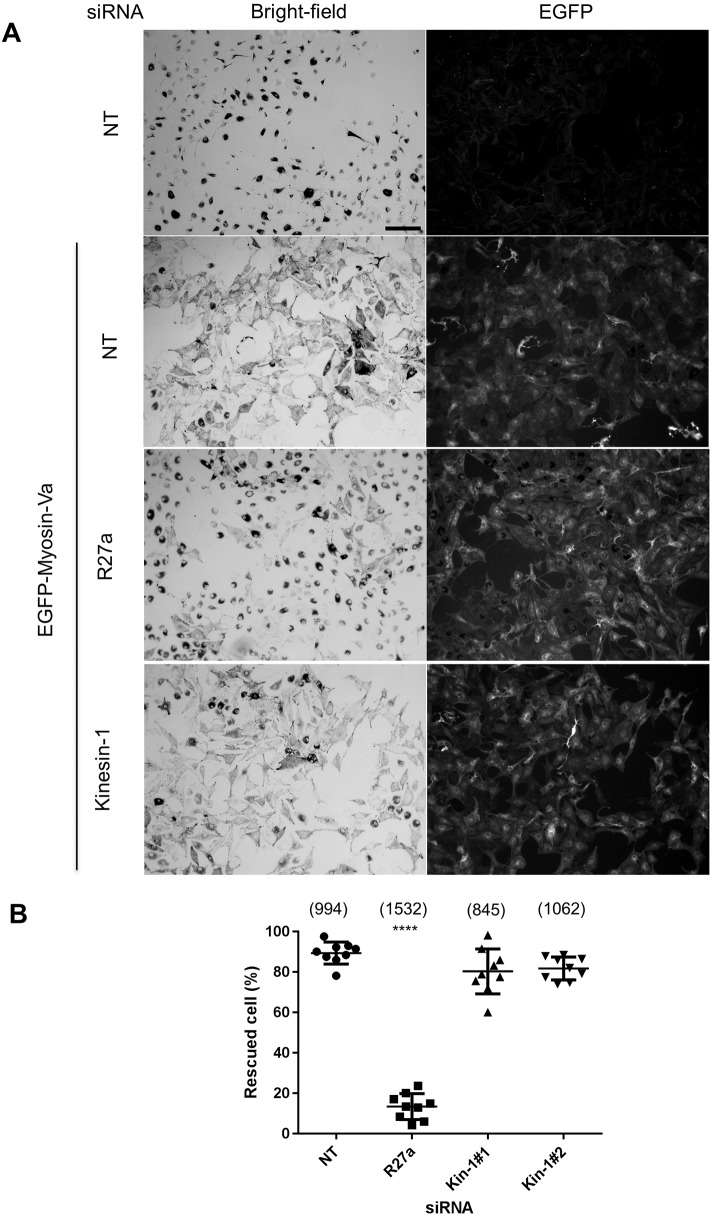
**Knockdown of Kif5b does not compromise myosin-Va-driven centrifugal melanosome transport.** melan-d1 cells were transfected with the indicated siRNA, then 72 h later infected with GFP–myosin-Va-expressing adenovirus, and fixed and stained with anti-GFP antibodies to reveal the expression of myosin-Va (as described the Materials and Methods). (A) Representative low-power bright-field and corresponding fluorescence images showing the subcellular distribution of melanosomes and expression of GFP–myosin-Va in fields of siRNA-transfected and/or GFP–myosin-Va-expressing melan-d1 cells. R27a, Rab27a siRNA. Scale bar: 100 µm. (B) A scatter plot showing the percentage of melanocytes manifesting perinuclear clustered melanosomes for each siRNA. Data are from 9 independent transfections, each performed in triplicate on different pools of cells. Plotted points represent the mean percentage of cells with perinuclear clustered melanosomes from each experiment (as described in the Materials and Methods). Bracketed numbers indicate the total number of cells analysed. Horizontal bars show the mean and 25^th^ and 75^th^ percentile of each population. ****P≤0.0001 relative to NT cells (one-way ANOVA); no other significant differences were observed.

Similarly, in the second centrifugal transport assay [myosin-Va-dependent dispersion of perinuclear melanosome clustering in myosin-Va-null (melan-d1) cells] siRNA-mediated depletion of Rab27a significantly reduced myosin-Va-dependent dispersion of perinuclear clustered melanosomes compared with that seen in control siRNA-transfected cells, while Kif5b-specific siRNA did not (Fig. 3A,B; the mean±s.d. percentage rescued cells is 13.45±6.426% for Rab27a siRNA versus 89.41±5.428% for cells with NT siRNA).

This further indicates that expression of Rab27a (and its effectors melanophilin and myosin-Va), but not Kif5b, is required for centrifugal melanosome transport in melanocytes (Fukuda, 2013; Hume et al., 2011). To confirm that the level of Kif5b knockdown was sufficient to block its function, we examined the distribution of mitochondria and GFP–SKIP in siRNA-transfected melanocytes. Previous studies revealed perinuclear accumulation of mitochondria in knockout cells and reduction in accumulation of GFP–SKIP at the cell periphery in Kif5b-depleted HeLa cells, indicating that Kif5b functions in centrifugal transport of mitochondria and GFP–SKIP (Dumont et al., 2010; Tanaka et al., 1998). Consistent with this, we observed perinuclear clustering of MitoTracker-labelled mitochondria and a reduction in the amount of peripheral localised GFP–SKIP in wild-type melanocytes transfected with Kif5b, but not Rab27a or control (NT), siRNA (Fig. 2B, Fig. S1C).

### Functional disruption of Rab1a affects the integrity of the Golgi but not centrifugal melanosome transport in melanocytes

**Fig. 4.**
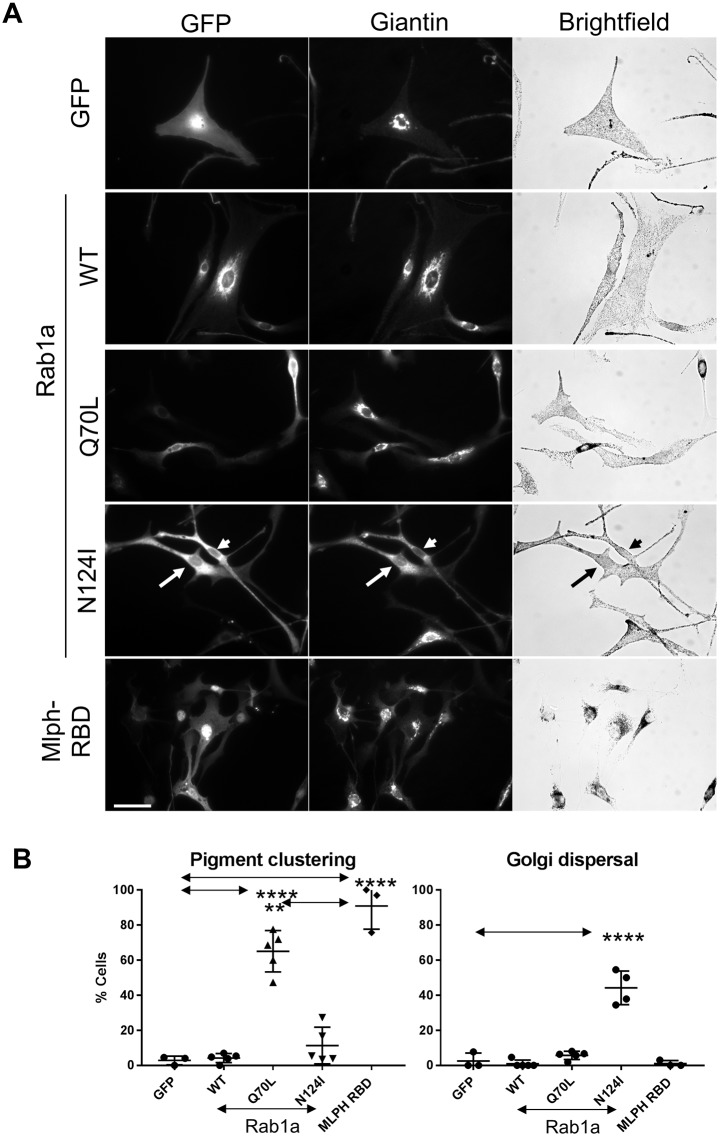
**Expression of a dominant-negative Rab1a^N124I^ mutant affects Golgi integrity but not melanosome distribution.** melan-a cells were infected with adenovirus vectors allowing expression of the indicated proteins, and were then fixed and stained with giantin-specific antibodies to reveal the structure of the Golgi (as described in the Materials and Methods). (A) Representative micrographs showing the distribution of GFP or GFP-tagged protein and giantin (Golgi) (fluorescence images), and melanosomes (bright-field) in infected melanocytes. For the Rab1a^N124I^ mutant, the arrow and arrowhead indicate the cells expressing mutant protein. Scale bar: 40 µm. (B) A scatter plot showing the percentage of melanocytes in which melanosomes were clustered in the perinuclear cytoplasm for cells expressing each protein. Data are from three (GFP and Mlph-RBD) or five (Rab1a and mutants) independent experiments. Plotted points represent the mean percentage of cells with perinuclear clustered melanosomes from each experiment (as described in the Materials and Methods). Total number of cells analysed were: GFP=73, Rab1a^WT^=153, Rab1a^Q70L^=196, Rab1a^N124I^=132 and Mlph-RBD=167. Horizontal bars show the mean and 25^th^ and 75^th^ percentile of each population. **P≤0.01, ****P≤0.0001 (one-way ANOVA); arrows indicate compared datasets, no other significant differences were observed.

To probe the role of Rab1a in melanocytes, we tested the effect of overexpression of mutants that alter its GDP–GTP exchange and GTPase activity, namely, Rab1a^N124I^ (nucleotide-free form) and Rab1a^Q70L^ (constitutively GTP-bound form). We found that expression of the inactive Rab1a^N124I^ mutants, but not wild-type or constitutively active (CA) Rab1a^Q70L^, resulted in fragmentation of the Golgi in a significant proportion of cells (44.22±9.584% for Rab1aN124I versus 2.564±4.441% for GFP; mean±s.d.). This is consistent with the reported ability of this mutant to disrupt Rab1a function in ER-to-Golgi trafficking (Wilson et al., 1994). Conversely, we found that expression of CA Rab1a^Q70L^, but not wild-type Rab1a or inactive Rab1a^N124I^, triggered melanosome clustering in a significant proportion of cells, albeit with significantly lower efficiency than the Rab27a-binding domain (RBD) of melanophilin (Mlph-RBD; 90.88±13.26% versus Rab1a^Q70L^ at 65.05±11.8%; Fig. 4).

Overall, these data do not support a positive role for Kif5b or Rab1a in driving melanosome dispersion. Indeed, the ability of the Rab1a^Q70L^ mutant to cause melanosome clustering, shown here and previously, support the possibility that Rab1a functions in a process that opposes centrifugal transport (Ishida et al., 2012, 2015).

### Forced targeting of Kif5b to melanosomes drives their MT-dependent dispersion

**Fig. 5.**
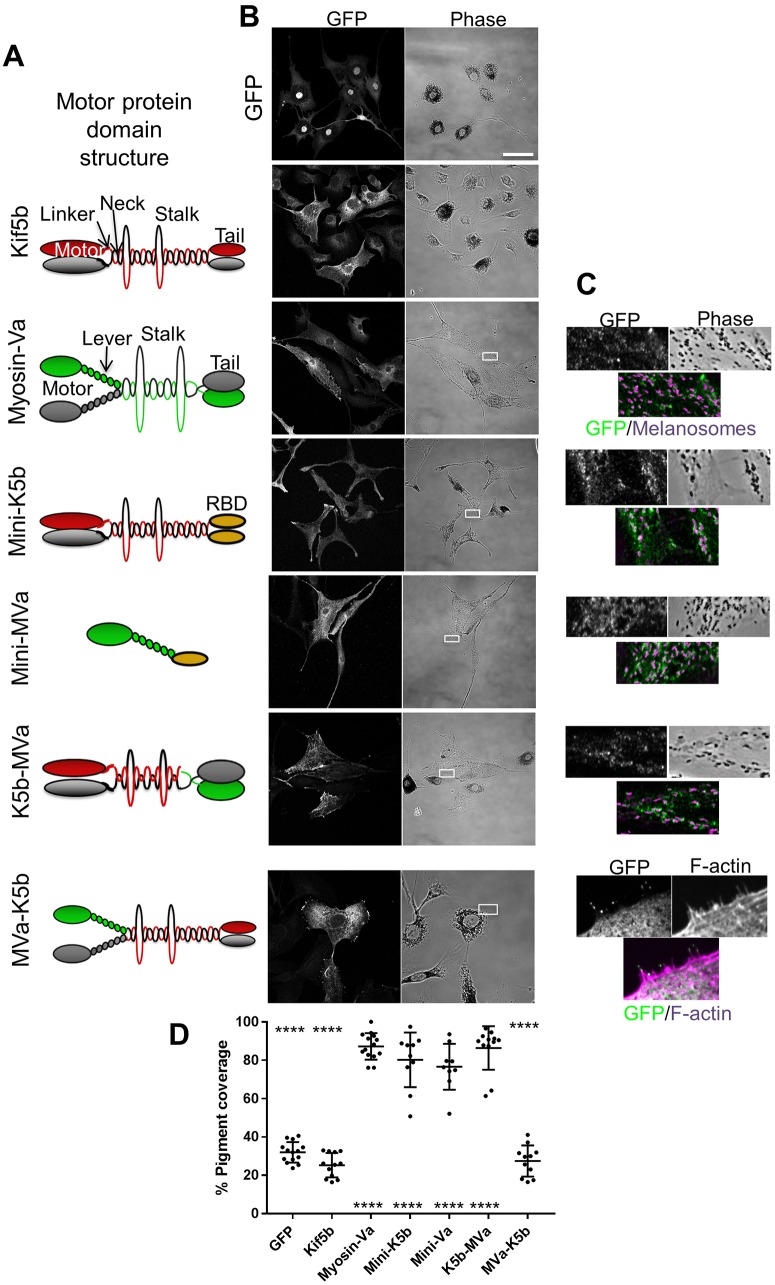
**Forced targeting of Kif5b to melanosomes can drive centrifugal melanosome transport in melanocytes.** melan-d1 cells were infected with adenovirus allowing expression of the indicated GFP fusion proteins then fixed 24 h later, processed for immunofluorescence and imaged using a confocal microscope (as described in the Materials and Methods). (A) Schematic representation of the domain organisation and expected oligomeric state of Kif5b (red and grey shapes), myosin-Va (green and grey shapes) and fusion proteins. RBD (orange shape) indicates the Rab27a-binding domain of murine Sytl2 [as described previously; Evans et al. (2014)]. (B) Pairs of confocal fluorescence and phase-contrast (transmitted light) images showing the distribution of GFP fusion proteins and melanosomes, respectively, in representative fields of infected cells. Scale bar: 50 µm. (C) High-magnification images of cells within the borders of the white boxes in lower magnification images phase-contrast images as shown in B, indicating the areas shown that highlight association between fusion proteins (green) and F-actin (for MVa–K5b) or melanosomes (other fusion proteins), both shown in magenta. (D) A scatter plot showing the pigment distribution as reported by percentage pigment coverage (pigment-filled area/total cell area), calculated as described previously (Hume et al., 2006). Horizontal bars show the median and 25th and 75th percentile of each population. The significance of differences in pigment coverage for each population compared with the GFP and wild-type myosin-Va are displayed below and above each scatter point. ****P≤0.0001 (one-way ANOVA); no other significant differences were observed. Data are from one of three independent experiments and are representative of the results of all experiments. Number of cells analysed; GFP=14, Kif5b=12, myosin-Va=14, mini-K5b=10, mini-Va=9, k5b-mva=12 and mVa-k5b=11.

As an alternative approach to investigate the function of Kif5b in transport, we tested the effect of GFP–Kif5b expression in myosin-Va-deficient melan-d1 cells. We found that GFP–Kif5b did not colocalise with, or disperse melanosomes to a significantly greater extent than GFP alone (mean pigment area=25.32±6.356% for GFP-Kif5b, versus=32.02±5.325% for GFP; mean±s.d.; Fig. 5A,B). This further suggests that Kif5b does not transport melanosomes.

One possible explanation for the lack of effect of Kif5b on transport is that there is an essential requirement for actin or myosin-Va in centrifugal transport that cannot be overcome by kinesin- and MT-dependent transport. Alternatively, melanosomes may have limited capacity to recruit Kif5b. To investigate these possibilities, we generated fusion proteins between Kif5b, myosin-Va and other melanosome-targeting proteins, and tested their function and localisation in melan-d1 cells. A similar approach, where fission yeast kinesin-7 (Tea2) was re-tasked to transport type V myosin (Myo52) cargo along MTs, has been used to show that transport on MTs can restore polarised growth and viability in cells lacking actin cables, which are the physiological tracks for Myo52 cargo (Lo Presti and Martin, 2011).

To target active Kif5b (an N-terminal fragment, amino acids 1–810, containing the motor, neck, linker and stalk regions, but lacking the C-terminus cargo-binding/regulatory tail) to melanosomes we used two strategies (Fig. 5A). First, we modified the previously described ‘mini-myosin’ (mini-Va) vector such that the myosin-Va S1 (motor and lever arm)-encoding fragment was replaced by active Kif5b. This protein, termed ‘mini-K5b’, targets to melanosomes via the RBD of Rab27a effector synaptotagmin-like protein 2 (Sytl2) (Fig. 5A–C). Secondly, we generated a Kif5b and myosin-Va (hereafter K5b–MVa) fusion protein, comprising (from the N-terminus): GFP, active Kif5b and the melanosome-binding tail fragment of myosin-Va (Strom, 2002; Fig. 5A). K5b–MVa, like myosin-Va, targets to melanosomes via the interaction of the myosin-Va tail with endogenous melanophilin. [Consistent with this K5b–MVa and full-length myosin-Va, in contrast to mini-Va and mini-K5b, were non-functional in melanophilin-deficient melan-ln cells (Fig. S2).] In parallel, we engineered the reciprocal MVa–K5b fusion protein comprising (from the N-terminus): (1) GFP, (2) the myosin-Va S1, and (3) motor-less Kif5b (i.e. a fragment that contains the neck, linker, stalk and cargo- and light-chain-binding tail) (Fig. 5A).

In line with previous studies, we found that full-length myosin-Va and mini-Va localised to, and significantly dispersed, melanosomes in melan-d1 cells compared with GFP alone (mean pigment area; myosin-Va 87.21±6.94% and mini-Va 76.59±11.97%; mean±s.d.; Fig. 5D; Evans et al., 2014). In contrast, MVa–K5b did not rescue melanosome distribution or localise to melanosomes, and instead localised to the actin-rich cell periphery, where it accumulated at the distal tips of filopodia-like extension, confirming the activity of the myosin-Va fragment (Fig. 5B,C). This is consistent with the lack of colocalisation between melanosomes and GFP–Kif5b and motor-less Kif5b (GFP–Kif5bΔ350) (Fig. S1A; Fig. 5B,C), and suggests that the function of Kif5b in melanosome transport is restricted by the limited capacity of melanosomes to recruit this motor.

In accordance with this, we observed that mini-K5b and K5b-mVa both localised to, and dispersed melanosomes with similar efficiency to myosin-Va and mini-Va (mean pigment area; mini-K5b=80.18±14.26% and K5b-MVa=86.37±11.33%). However, in contrast to myosin-Va and mini-Va, and in line with the role of Kif5b as a MT motor, their activity was dependent upon MT integrity (mean pigment area in MT-depleted cells; mini-K5b=46.84±12.05%, K5b-MVa=47.82±3.271%, GFP=36.45±9.469% versus myosin-Va=78.42±9.144% and mini-Va=57.5±16.55%; Fig. S3). Further supporting the MT dependence of mini-K5b- and K5b-MVa-driven transport, we observed that, in many cells expressing these proteins, melanosomes accumulated in the tips of dendrites and were relatively depleted in the central cell cytoplasm (see Fig. S4A for an example). This is consistent with the role of Kif5b as a plus-end-directed motor and the peripheral distribution of MT plus-ends in melanocytes.

Collectively, these data indicate that Kif5b can disperse melanosomes with similar efficiency to myosin-Va, but that its capacity to do so is restricted by the limited ability of its cargo-binding tail to associate with melanosomes.

## DISCUSSION

Here, we probed the role of Kif5b and Rab1a in centrifugal melanosome transport in melanocytes, and revealed two main findings.

First, we were unable to find evidence that Kif5b or Rab1a contribute to centrifugal melanosome transport. By using several pairs of siRNA oligonucleotides, we found that knockdown of Kif5b (the most abundant kinesin-1 heavy chain in melanocytes) did not affect the usual dispersed distribution of melanosomes in wild-type melanocytes or the ability of GFP–myosin-Va to rescue melanosome clustering in myosin-Va-deficient melan-d1 cells, both read-outs of centrifugal transport (Figs 2, 3). Meanwhile, disruption of Rab1a function by expression of the dominant-negative mutant Rab1a^N124I^ did not affect melanosome distribution in wild-type melanocytes, arguing against its role in promoting their centrifugal transport (Fig. 4). The latter possibility is further undermined by the observation that expression of the constitutively active Rab1a^Q70L^ caused melanosome clustering in wild-type cells (Fig. 4; Ishida et al., 2012). Taken together, with the lack of obvious association between Kif5b or Rab1a and melanosomes (Fig. 1), these data suggest that Kif5b and Rab1a are less important than myosin-Va to centrifugal transport. This chimes with our previously published data showing: (1) that MT integrity is not essential for centrifugal transport driven by myosin-Va and dynamic actin, and (2) that only 10% of melanosome movements in wild-type melanocytes were MT dependent (Evans et al., 2014; Hume et al., 2011). Similar results have been reported regarding the contribution of MTs to centrifugal transport in amphibian melanophores, indicating that myosin-Va and F-actin likely play an important role in melanosome transport in multiple species (Gross et al., 2002; Rogers and Gelfand, 1998; Schliwa and Euteneuer, 1978). Furthermore, in preliminary experiments, we found that transfection of wild-type melanocytes with siRNA pools targeted against each of the 46 mouse kinesin heavy chains genes had no detectable effect on melanosome dispersion (A.N.H., I. Meschede and M. Seabra, unpublished observations). Taken together, the data presented here and previously suggests that MTs and plus-end-directed kinesins (e.g. Kif5b) are not the main drivers of centrifugal transport.

Secondly, we found that the role of Kif5b in transport is limited by the capacity of melanosomes to recruit the motor. We saw that although an active Kif5b motor and stalk fragment could disperse melanosomes in melan-d1 cells when artificially targeted there through replacement of the endogenous tail with the Rab27a-binding domain of Sytl2a (mini-K5b) or the melanophilin-binding tail of myosin-Va (K5b–MVa), this was not the case for proteins containing the endogenous Kif5b tail (Figs 1 and 5; Fig. S1A). The latter finding is consistent with the negative results of our functional studies of Kif5b and Rab1a in melanosome transport, and suggests that a dearth of targeting factors limits Kif5b function in this process (Figs 2–4).

Interestingly, in many cases, expression of artificially melanosome-targeted Kif5b protein resulted in hyper-accumulation of melanosomes in the peripheral cytoplasm (Fig. S4A). A similar phenomenon, termed ‘peripheral melanosome aggregation’, was observed in other studies in a subset (∼25%) of melan-a cells in which Rab1a^Q70L^ and SKIP were co-expressed (Ishida et al., 2015). In contrast, as shown here and previously, individual expression of Rab1a^Q70L^ and SKIP causes melanosome clustering and dispersion of lysosomes, respectively (Ishida et al., 2012, 2015; Rosa-Ferreira and Munro, 2011; Dumont et al., 2010) (Fig. 4; Fig. S1B). In lysosome dispersion, SKIP acts as an adaptor allowing Arl8b to recruit Kif5b to lysosomes. Recent data, showing that SKIP interacts with Rab1a^Q70L^, suggests that a similar mechanism underlies SKIP-driven dispersion of the Golgi (i.e. SKIP acting as an adaptor allowing active Rab1a to recruit Kif5b to the Golgi). Although the mechanism of Rab1a^Q70L^-mediated melanosome clustering is unclear, it was previously shown (and confirmed here) that Rab1a^Q70L^ can associate with melanosomes and thus could disperse melanosomes by recruiting SKIP and Kif5b (Ishida et al., 2012, 2015) (Fig. S4B). However, the observation that dual SKIP and Rab1a^Q70L^ expression is required for dispersion suggests that physiological levels of SKIP in melanocytes are insufficient to allow melanosomal active Rab1a to recruit enough Kif5b to disperse melanosomes. Conversely, the finding that SKIP expressed alone does not localise to, or affect the distribution of melanosomes, suggests that melanosomes contain low levels of active Rab1a compared with those seen in the Golgi. This further supports the idea that the Rab1a/SKIP/KLC2/Kif5b axis does not play a significant role in transport in the absence of overexpression.

Finally, how do we reconcile our conclusion that Kif5b is not a significant melanosome transporter with previous studies, which proposed that Kif5b, recruited by Rab1a, SKIP and KLC2, regulates MT-dependent centrifugal melanosome transport (Ishida et al., 2012, 2015)? One possibility is that the level of knockdown achieved in our study, although sufficient to affect transport of mitochondria and GFP–SKIP, was insufficient to affect Kif5b function in melanosome transport. This might be the case if relatively few motors are required to move melanosomes compared with other cargo. Another possibility is that the cells used here and previously (Ishida et al., 2012, 2015) differ in their ability to recruit Kif5b to melanosomes. While we cannot exclude this possibility, it is noteworthy that neither of the previous studies directly tested the ability of Kif5b to associate with melanosomes nor did they directly test the requirement for Kif5b in melanosome dispersion. Leading from this, while we agree that melanosome clustering seen in the previous studies in cells depleted for Kif5b, SKIP, Rab1a and KLC2 is consistent with a role for these proteins in MT-dependent centrifugal transport, we consider that caveats remain regarding this conclusion. Firstly, it is not clear that the observed clustering is due to defects in MT-dependent transport rather than an indirect effect of disrupting the F-actin and/or myosin-Va transport system. We suggest that to conclude that results are a direct effect of defects in MT-dependent transport, it would be essential to test whether the rescue of clustering, i.e. centrifugal melanosome transport, is MT dependent. Secondly, if Kif5b is an essential regulator of centrifugal melanosomes transport, it is not clear why clustering was observed in only 20–40% of depleted cells. In contrast, depletion of Rab27a results in clustering in 80–100% of transfected cells (Figs 2–3). Indeed, the low proportion of clustering observed in Kif5b-depleted cells is particularly surprising given that the depletion of the siRNA target proteins seen in western blotting appears to be greater than 20–40% (Ishida et al., 2015). Thirdly, we were unable to reproduce the melanosome clustering observed in those previous studies using the same cell line and the same oligonucleotides. In conclusion, while we cannot totally exclude a role for Kif5b in melanosome transport, our data presented here and previously do not support the idea that is has an essential role in this process.

## MATERIALS AND METHODS

### Cell culture and transfection

Cultures of immortal melan-a, melan-d1 and melan-ln melanocytes were maintained, and transfected with plasmid and siRNA oligonucleotides, as described previously (Evans et al., 2014; Hume et al., 2007). For depletion of MTs, cells were cultured in growth medium supplemented with 10 µM nocodazole (Sigma, product code M1404). For details of the sequences of siRNA used in this study see Table S1.

### Plasmid and virus constructs

Generation of plasmid vectors pEGFPC3-Rab27a and pEGFPC3-Rab1a and adenovirus allowing expression of GFP–Rab27a, GFP–Rab1a and GFP–myosin-Va (melanocyte isoform) in melanocytes has been previously described (Evans et al., 2014; Hume et al., 2001). pENTR-GFPC2-KIf5bΔ350 and pENTR-GFPC2-SKIP allowing expression of GFP–KIf5bΔ350 and GFP–SKIP were generated in the course of this study. pEYFPN1-KIF5B, allowing expression of human Kif5b at the N-terminus of YFP, was generated by Dr Chen Gu (Ohio State University, OH) as described previously (Gu et al., 2006), and gifted to us by Dr Stefan Linder (Hamburg, Germany). pENTR-myc-SKIP allowing expression of SKIP tagged at the N-terminus with the Myc epitope was gifted to us by Dr Sean Munro (MRC-LMB, Cambridge, UK). To investigate the role of Kif5b and Rab1a in melanosome transport and its targeting to melanosomes, adenovirus vectors were generated that allow expression of the following fusion proteins in melanocytes: GFP–Kif5b, mini-K5b, K5b–MVa, MVa–K5b, GFP–Rab1a, GFP–Rab1a^Q70L^ and GFP–Rab1a^N124I^, as described previously (Hume et al., 2006). Adenoviruses allowing expression of melanophilin–RBD, mini-Va and full-length myosin-Va, containing the melanocyte-specific exons D and F, were as previously described (Evans et al., 2014; Hume et al., 2006). Primer sequences and further details of the cloning procedures used are available on request.

### Immunoblotting

Immunoblotting was performed as described previously (Hume et al., 2007) using goat anti-Kif5b (Everest Biotech EB05492; 1:1000), goat anti-GAPDH (Sicgen Ab0049-200; 1:5000) and rabbit anti-tyrosinase (PEP7, a kind gift from Dr Vincent Hearing, NCI-NIH, USA; 1:1000) primary antibodies, and IRDye 800CW-conjugated secondary antibodies (Odyssey 926-32214; 1:10,000). Signal was detected using a Li-Cor infra-red scanner (Odyssey).

### Microscopy and image analysis

Cells for immunofluorescence were paraformaldehyde fixed and stained, and fluorescence and transmitted-light images of melanocytes were then collected using a Zeiss LSM710 confocal microscope fitted with a 63×1.4 NA oil immersion Apochromat lens. All images presented are single sections in the *z*-plane. Antibodies and stains were used as indicated: mouse monoclonal anti-GFP antibody (Roche 11814460001; 1:300), rabbit anti-tyrosinase antibody (kindly supplied by Dr Vincent Hearing; 1:100), rabbit-anti-giantin antibody (Abcam 24586; 1:1000), mouse-anti-tubulin clone DM1a antibody (Calbiochem cp06; 1:100), goat anti-mouse-IgG conjugated to Alexa Fluor 488 and goat anti-rabbit-IgG conjugated to Alexa Fluor 568 secondary antibodies (Invitrogen A-11001 and A-11011; both 1:500). For live-cell visualisation of mitochondria, melanocytes in µ-slide 8-well glass-bottomed chamber slides (Ibidi IB-80827) were incubated for 30 min with medium containing 200 nM MitoTracker® Red CMXRos (Invitrogen M7512). For analysis of melanosome clustering, siRNA transfections were carried out in triplicate (i.e. three wells of a 24-well plate for each siRNA in each experiment). After 72 h phase-contrast images of three different randomly-selected fields of cells in each well were captured using Axiovision 4.8 software associated with a Zeiss Axiovert 100S inverted microscope fitted with a 10× objective and an Axiocam CCD camera. Images were then randomised, and the number of cells with clustered melanosomes in each field was counted by a researcher blinded to the identity of the siRNA transfected into each field of cells. Cells in which pigmented melanosomes were contained within the perinuclear cytoplasm (<50% of the total cytoplasmic area) were defined as containing clustered melanosomes. Measurement of the function of chimeric Kif5b fusion proteins in melanosome transport (Fig. 5) was based on manual measurement of the proportion of cell area occupied by pigmented melanosomes as previously described (Hume et al., 2006).

## References

[JCS186064C1] Aizawa M. and Fukuda M. (2015). Small GTPase Rab2B and its specific binding protein Golgi-associated Rab2B interactor-like 4 (GARI-L4) regulate Golgi morphology. *J. Biol. Chem.* 290, 22250-22261. 10.1074/jbc.M115.669242 26209634 PMC4571976

[JCS186064C2] Alberts B., Johnson A., Lewis J., Raff M., Roberts K. and Walter P. (2008). *Molecular Biology of the Cell.* Garland Science, New York.

[JCS186064C3] Barlan K., Rossow M. J. and Gelfand V. I. (2013). The journey of the organelle: teamwork and regulation in intracellular transport. *Curr. Opin. Cell Biol.* 25, 483-488. 10.1016/j.ceb.2013.02.018 23510681 PMC3723706

[JCS186064C4] Byers H. R., Yaar M., Eller M. S., Jalbert N. L. and Gilchrest B. A. (2000). Role of cytoplasmic dynein in melanosome transport in human melanocytes. *J. Invest. Dermatol.* 114, 990-997. 10.1046/j.1523-1747.2000.00957.x 10771482

[JCS186064C5] Dumont A., Boucrot E., Drevensek S., Daire V., Gorvel J.-P., Poüs C., Holden D. W. and Méresse S. (2010). SKIP, the host target of the Salmonella virulence factor SifA, promotes kinesin-1-dependent vacuolar membrane exchanges. *Traffic* 11, 899-911. 10.1111/j.1600-0854.2010.01069.x 20406420

[JCS186064C6] Evans R. D., Robinson C., Briggs D. A., Tooth D. J., Ramalho J. S., Cantero M., Montoliu L., Patel S., Sviderskaya E. V. and Hume A. N. (2014). Myosin-Va and dynamic actin oppose microtubules to drive long-range organelle transport. *Curr. Biol.* 24, 1743-1750. 10.1016/j.cub.2014.06.019 25065759 PMC4131108

[JCS186064C7] Fukuda M. (2013). Rab27 effectors, pleiotropic regulators in secretory pathways. *Traffic* 14, 949-963. 10.1111/tra.12083 23678941

[JCS186064C8] Fukuda M., Kuroda T. S. and Mikoshiba K. (2002). Slac2-a/melanophilin, the missing link between Rab27 and myosin Va: implications of a tripartite protein complex for melanosome transport. *J. Biol. Chem.* 277, 12432-12436. 10.1074/jbc.C200005200 11856727

[JCS186064C9] Goode B. L., Drubin D. G. and Barnes G. (2000). Functional cooperation between the microtubule and actin cytoskeletons. *Curr. Opin. Cell Biol.* 12, 63-71. 10.1016/S0955-0674(99)00058-7 10679357

[JCS186064C10] Gross S. P., Tuma M. C., Deacon S. W., Serpinskaya A. S., Reilein A. R. and Gelfand V. I. (2002). Interactions and regulation of molecular motors in Xenopus melanophores. *J. Cell Biol.* 156, 855-865. 10.1083/jcb.200105055 11864991 PMC2173315

[JCS186064C11] Gu C., Zhou W., Puthenveedu M. A., Xu M., Jan Y. N. and Jan L. Y. (2006). The microtubule plus-end tracking protein EB1 is required for Kv1 voltage-gated K+ channel axonal targeting. *Neuron* 52, 803-816. 10.1016/j.neuron.2006.10.022 17145502

[JCS186064C12] Gupta V., Palmer K. J., Spence P., Hudson A. and Stephens D. J. (2008). Kinesin-1 (uKHC/KIF5B) is required for bidirectional motility of ER exit sites and efficient ER-to-Golgi transport. *Traffic* 9, 1850-1866. 10.1111/j.1600-0854.2008.00811.x 18817524

[JCS186064C13] Hammer J. A. III and Sellers J. R. (2012). Walking to work: roles for class V myosins as cargo transporters. *Nat. Rev. Mol. Cell Biol.* 13, 13-26. 10.1038/nrm3248 22146746

[JCS186064C14] Hara M., Yaar M., Byers H. R., Goukassian D., Fine R. E., Gonsalves J. and Gilchrest B. A. (2000). Kinesin participates in melanosomal movement along melanocyte dendrites. *J. Invest. Dermatol.* 114, 438-443. 10.1046/j.1523-1747.2000.00894.x 10692101

[JCS186064C15] Hirokawa N., Noda Y., Tanaka Y. and Niwa S. (2009). Kinesin superfamily motor proteins and intracellular transport. *Nat. Rev. Mol. Cell Biol.* 10, 682-696. 10.1038/nrm2774 19773780

[JCS186064C16] Holubcová Z., Howard G. and Schuh M. (2013). Vesicles modulate an actin network for asymmetric spindle positioning. *Nat. Cell Biol.* 15, 937-947. 10.1038/ncb2802 23873150 PMC3797517

[JCS186064C17] Hume A. N. and Seabra M. C. (2011). Melanosomes on the move: a model to understand organelle dynamics. *Biochem. Soc. Trans.* 39, 1191-1196. 10.1042/BST0391191 21936787

[JCS186064C18] Hume A. N., Collinson L. M., Rapak A., Gomes A. Q., Hopkins C. R. and Seabra M. C. (2001). Rab27a regulates the peripheral distribution of melanosomes in melanocytes. *J. Cell Biol.* 152, 795-808. 10.1083/jcb.152.4.795 11266470 PMC2195786

[JCS186064C19] Hume A. N., Tarafder A. K., Ramalho J. S., Sviderskaya E. V. and Seabra M. C. (2006). A coiled-coil domain of melanophilin is essential for Myosin Va recruitment and melanosome transport in melanocytes. *Mol. Biol. Cell* 17, 4720-4735. 10.1091/mbc.E06-05-0457 16914517 PMC1635380

[JCS186064C20] Hume A. N., Ushakov D. S., Tarafder A. K., Ferenczi M. A. and Seabra M. C. (2007). Rab27a and MyoVa are the primary Mlph interactors regulating melanosome transport in melanocytes. *J. Cell Sci.* 120, 3111-3122. 10.1242/jcs.010207 17698919

[JCS186064C21] Hume A. N., Wilson M. S., Ushakov D. S., Ferenczi M. A. and Seabra M. C. (2011). Semi-automated analysis of organelle movement and membrane content: understanding rab-motor complex transport function. *Traffic* 12, 1686-1701. 10.1111/j.1600-0854.2011.01283.x 21920004 PMC3264752

[JCS186064C22] Ishida M., Ohbayashi N., Maruta Y., Ebata Y. and Fukuda M. (2012). Functional involvement of Rab1A in microtubule-dependent anterograde melanosome transport in melanocytes. *J. Cell Sci.* 125, 5177-5187. 10.1242/jcs.109314 22854043

[JCS186064C23] Ishida M., Ohbayashi N. and Fukuda M. (2015). Rab1A regulates anterograde melanosome transport by recruiting kinesin-1 to melanosomes through interaction with SKIP. *Sci. Rep.* 5, 8238 10.1038/srep08238 25649263 PMC4316160

[JCS186064C24] Kapitein L. C., Van Bergeijk P., Lipka J., Keijzer N., Wulf P. S., Katrukha E. A., Akhmanova A. and Hoogenraad C. C. (2013). Myosin-V opposes microtubule-based cargo transport and drives directional motility on cortical actin. *Curr. Biol.* 23, 828-834. 10.1016/j.cub.2013.03.068 23602478

[JCS186064C25] Kardon J. R. and Vale R. D. (2009). Regulators of the cytoplasmic dynein motor. *Nat. Rev. Mol. Cell Biol.* 10, 854-865. 10.1038/nrm2804 19935668 PMC3394690

[JCS186064C26] Linstedt A. D. and Hauri H. P. (1993). Giantin, a novel conserved Golgi membrane protein containing a cytoplasmic domain of at least 350 kDa. *Mol. Biol. Cell* 4, 679-693. 10.1091/mbc.4.7.679 7691276 PMC300978

[JCS186064C27] Lo Presti L. and Martin S. G. (2011). Shaping fission yeast cells by rerouting actin-based transport on microtubules. *Curr. Biol.* 21, 2064-2069. 10.1016/j.cub.2011.10.033 22137473

[JCS186064C28] Marchesin V., Castro-Castro A., Lodillinsky C., Castagnino A., Cyrta J., Bonsang-Kitzis H., Fuhrmann L., Irondelle M., Infante E., Montagnac G. et al. (2015). ARF6-JIP3/4 regulate endosomal tubules for MT1-MMP exocytosis in cancer invasion. *J. Cell Biol.* 211, 339-358. 10.1083/jcb.201506002 26504170 PMC4621834

[JCS186064C29] Matsui T., Ohbayashi N. and Fukuda M. (2012). The Rab interacting lysosomal protein (RILP) homology domain functions as a novel effector domain for small GTPase Rab36: Rab36 regulates retrograde melanosome transport in melanocytes. *J. Biol. Chem.* 287, 28619-28631. 10.1074/jbc.M112.370544 22740695 PMC3436592

[JCS186064C30] Navone F., Niclas J., Hom-Booher N., Sparks L., Bernstein H. D., Mccaffrey G. and Vale R. D. (1992). Cloning and expression of a human kinesin heavy chain gene: interaction of the COOH-terminal domain with cytoplasmic microtubules in transfected CV-1 cells. *J. Cell Biol.* 117, 1263-1275. 10.1083/jcb.117.6.1263 1607388 PMC2289507

[JCS186064C31] Nuoffer C., Davidson H. W., Matteson J., Meinkoth J. and Balch W. E. (1994). A GDP-bound of rab1 inhibits protein export from the endoplasmic reticulum and transport between Golgi compartments. *J. Cell Biol.* 125, 225-237. 10.1083/jcb.125.2.225 8163542 PMC2120023

[JCS186064C32] Ohbayashi N., Maruta Y., Ishida M. and Fukuda M. (2012). Melanoregulin regulates retrograde melanosome transport through interaction with the RILP-p150Glued complex in melanocytes. *J. Cell Sci.* 125, 1508-1518. 10.1242/jcs.094185 22275436

[JCS186064C33] Provance D. W. Jr, Wei M., Ipe V. and Mercer J. A. (1996). Cultured melanocytes from dilute mutant mice exhibit dendritic morphology and altered melanosome distribution. *Proc. Natl. Acad. Sci. USA* 93, 14554-14558. 10.1073/pnas.93.25.14554 8962090 PMC26171

[JCS186064C34] Pruyne D., Legesse-Miller A., Gao L., Dong Y. and Bretscher A. (2004). Mechanisms of polarized growth and organelle segregation in yeast. *Annu. Rev. Cell Dev. Biol.* 20, 559-591. 10.1146/annurev.cellbio.20.010403.103108 15473852

[JCS186064C35] Raposo G. and Marks M. S. (2007). Melanosomes--dark organelles enlighten endosomal membrane transport. *Nat. Rev. Mol. Cell Biol.* 8, 786-797. 10.1038/nrm2258 17878918 PMC2786984

[JCS186064C36] Rogers S. L. and Gelfand V. I. (1998). Myosin cooperates with microtubule motors during organelle transport in melanophores. *Curr. Biol.* 8, 161-164. 10.1016/S0960-9822(98)70063-6 9443916

[JCS186064C37] Rosa-Ferreira C. and Munro S. (2011). Arl8 and SKIP act together to link lysosomes to kinesin-1. *Dev. Cell* 21, 1171-1178. 10.1016/j.devcel.2011.10.007 22172677 PMC3240744

[JCS186064C38] Ross J. L., Ali M. Y. and Warshaw D. M. (2008). Cargo transport: molecular motors navigate a complex cytoskeleton. *Curr. Opin. Cell Biol.* 20, 41-47. 10.1016/j.ceb.2007.11.006 18226515 PMC2688467

[JCS186064C39] Schliwa M. and Euteneuer U. (1978). A microtuble-independent component may be involved in granule transport in pigment cells. *Nature* 273, 556-558. 10.1038/273556a0 351407

[JCS186064C40] Schuh M. (2011). An actin-dependent mechanism for long-range vesicle transport. *Nat. Cell Biol.* 13, 1431-1436. 10.1038/ncb2353 21983562 PMC3783939

[JCS186064C41] Segev N., Mulholland J. and Botstein D. (1988). The yeast GTP-binding YPT1 protein and a mammalian counterpart are associated with the secretion machinery. *Cell* 52, 915-924. 10.1016/0092-8674(88)90433-3 3127057

[JCS186064C41a] Strom M., Hume A. N., Tarafder A. K., Barkagianni E. and Seabra M. C. (2002). A family of Rab27-binding proteins. Melanophilin links Rab27a and myosin Va function in melanosome transport. *J. Biol. Chem.* 277, 25423-25430. 10.1074/jbc.M202574200 11980908

[JCS186064C42] Tanaka Y., Kanai Y., Okada Y., Nonaka S., Takeda S., Harada A. and Hirokawa N. (1998). Targeted disruption of mouse conventional kinesin heavy chain, kif5B, results in abnormal perinuclear clustering of mitochondria. *Cell* 93, 1147-1158. 10.1016/S0092-8674(00)81459-2 9657148

[JCS186064C43] Tisdale E. J., Bourne J. R., Khosravi-Far R., Der C. J. and Balch W. E. (1992). GTP-binding mutants of rab1 and rab2 are potent inhibitors of vesicular transport from the endoplasmic reticulum to the Golgi complex. *J. Cell Biol.* 119, 749-761. 10.1083/jcb.119.4.749 1429835 PMC2289685

[JCS186064C44] Vancoillie G., Lambert J., Mulder A., Koerten H. K., Mommaas A. M., Van Oostveldt P. and Naeyaert J.-M. (2000a). Cytoplasmic dynein colocalizes with melanosomes in normal human melanocytes. *Br. J. Dermatol.* 143, 298-306. 10.1046/j.1365-2133.2000.03654.x 10951136

[JCS186064C45] Vancoillie G., Lambert J., Mulder A., Koerten H. K., Mommaas A. M., Van Oostveldt P. and Naeyaert J. M. (2000b). Kinesin and kinectin can associate with the melanosomal surface and form a link with microtubules in normal human melanocytes. *J. Invest. Dermatol.* 114, 421-429. 10.1038/jid.2000.3 10692099

[JCS186064C46] Wei Q., Wu X. and Hammer J. A. III (1997). The predominant defect in dilute melanocytes is in melanosome distribution and not cell shape, supporting a role for myosin V in melanosome transport. *J. Muscle Res. Cell Motil.* 18, 517-527. 10.1023/A:1018659117569 9350005

[JCS186064C47] Wilson B. S., Nuoffer C., Meinkoth J. L., McCaffery M., Feramisco J. R., Balch W. E. and Farquhar M. G. (1994). A Rab1 mutant affecting guanine nucleotide exchange promotes disassembly of the Golgi apparatus. *J. Cell Biol* 125, 557-571. 10.1083/jcb.125.3.557 8175881 PMC2119990

[JCS186064C48] Woolner S. and Bement W. M. (2009). Unconventional myosins acting unconventionally. *Trends Cell Biol.* 19, 245-252. 10.1016/j.tcb.2009.03.003 19406643 PMC4878029

[JCS186064C49] Wu X., Bowers B., Rao K., Wei Q. and Hammer J. A. III (1998). Visualization of melanosome dynamics within wild-type and dilute melanocytes suggests a paradigm for myosin V function In vivo. *J. Cell Biol.* 143, 1899-1918. 10.1083/jcb.143.7.1899 9864363 PMC2175227

[JCS186064C50] Wu X. S., Rao K., Zhang H., Wang F., Sellers J. R., Matesic L. E., Copeland N. G., Jenkins N. A. and Hammer J. A. III (2002). Identification of an organelle receptor for myosin-Va. *Nat. Cell Biol.* 4, 271-278. 10.1038/ncb760 11887186

[JCS186064C51] Wu X. S., Tsan G. L. and Hammer J. A. III (2005). Melanophilin and myosin Va track the microtubule plus end on EB1. *J. Cell Biol.* 171, 201-207. 10.1083/jcb.200503028 16247022 PMC2171176

